# Biodegradation of Decabromodiphenyl Ether (BDE-209) by Crude Enzyme Extract from *Pseudomonas aeruginosa*

**DOI:** 10.3390/ijerph120911829

**Published:** 2015-09-18

**Authors:** Yu Liu, Ai-Jun Gong, Li-Na Qiu, Jing-Rui Li, Fu-Kai Li

**Affiliations:** 1 School of Chemistry and Biological Engineering, University of Science and Technology Beijing, Beijing 100083, China; E-Mails: liuyu2007@yahoo.com (Y.L.); qiulina@ustb.edu.cn (L.-N.Q.); l460919452@163.com (J.-R.L.); FK_liHarrison@163.com (F.-K.L.); 2 Dapartment of Biotechnology, Daqing Branch of Heilongjiang Academy of Science, Daqing 163319, China

**Keywords:** BDE-209, biodegradation, *Pseudomonas aeruginosa*, crude enzyme extract, biodegradation mechanism

## Abstract

The biodegradation effect and mechanism of decabromodiphenyl ether (BDE-209) by crude enzyme extract from *Pseudomonas aeruginosa* were investigated. The results demonstrated that crude enzyme extract exhibited obviously higher degradation efficiency and shorter biodegradation time than *Pseudomonas aeruginosa* itself. Under the optimum conditions of pH 9.0, 35 °C and protein content of 2000 mg/L, 92.77% of the initial BDE-209 (20 mg/L) was degraded after 5 h. A BDE-209 biodegradation pathway was proposed on the basis of the biodegradation products identified by GC-MS analysis. The biodegradation mechanism showed that crude enzyme extract degraded BDE-209 into lower brominated PBDEs and OH-PBDEs through debromination and hydroxylation of the aromatic rings.

## 1. Introduction

As global environmental contaminants, polybrominated diphenyl ethers (PBDEs) have 209 congeners that are distinguished by the number and location of bromine atoms in the benzene ring [1]. PBDEs are a major type of brominated flame retardants (BFRs) that have been widely used in a variety of household items and consumer products to delay the ignition of materials, such as plastics, textiles, foams and electronic (E&E) equipment [2]. Due to their stable chemical properties, PBDEs are persistent, bioaccumulative and toxic to environment [3]. Due to their wide-spread use, PBDEs have been detected in the atmosphere, water, soil, sediment and organisms all over the world, even in the Antarctica and Arctic. Moreover, their levels in human and animal bodies are increasing [4].

PBDEs have mainly three commercial formulations: penta-, octa- and deca-BDE. Due to their character and toxicity, penta-BDE and octa-BDE were listed at the Stockholm Convention in 2009 and are thus banned throughout the world [5]. The deca-BDE mixture, which is composed of mainly deca-BDE (BDE-209), was banned for use in E&E products in 2008 but was exempted from the directive [6]. It was the only commercial PBDE mixture that was still allowed to be produced, so its commercial production was rising [7]. Until now, despite that BDE-209 was banned in Europe and parts of the United States, a legacy of products still continue to release it into the environment from a wide range of sources [8]. For the past few decades, BDE-209 was considered less toxic than the lower brominated congeners, but now its potential risk to human health must not be overlooked. Studies have shown that BDE-209 has neurotoxicity, liver toxicity, endocrine toxicity, reproductive toxicity and potentially carcinogenicity effects [9]. Therefore, BDE-209 has attracted great attention of scientists at home and abroad, and it is extremely urgent to develop efficient and environmentally-friendly degradation methods to treat the heavy BDE-209 contaminants. So far, several degradation methods of BDE-209 have been reported, such as ultraviolet photolysis, oxidative degradation, reductive degradation, and biodegradation [10,11,12,13]. Among these, biodegradation has become popular because it has advantages of low cost, high removal efficiency, and non-pollution [14]. Weifang city of Shandong province has developed into the biggest base of BFRs in China, produces and sells BDE-209 to many areas of Asia [15]. In 2014, our laboratory successfully isolated a BDE-209 degrading strain (degradation efficiency 85%) from sludge of a BFRs factory in Weifang city, identified and named *Pseudomonas aeruginosa* LY11 (“LY11” represented the strain number). *Pseudomonas aeruginosa* is a common bacteria in the environment, Shi *et al.* used it and its crude enzyme to degrade BDE-209 [16].

Up to now, the reports on the biodegradation of BDE-209 are mostly focused on microorganism degradation. These microorganisms exhibit a good degradation effect of BDE-209, but there still exist certain problems, for example, it time consuming and application limiting. Thus, it has significant industrialization prospects. As a matter of fact, degradation enzymes play a key role in microorganism degradation; they can specifically bind substrate and catalyze degradation, and their degradation effect is far better than the microorganism itself [17]. Besides, they cannot cause secondary pollution, and can operate over a wider range of pollutant concentrations. Therefore, degradation enzymes have great potential for exploitation. Crude enzyme extract consist of intracellular enzyme mixtures, and degradation enzymes are contained within. Usually, degradation enzymes are obtained by separating crude enzyme extract, but this procedure will greatly increase the production costs. In order to reduce costs, crude enzyme extract was recently developed by some researchers and was found to exhibit a good degradation effect. For instance, Asgher *et al.* obtained crude enzyme extract from *Schyzohyllum commune* IBL-06 and used it for bioremediation of textile industry effluents [18]. So far, the biodegradation of BDE-209 by crude enzyme extract from *Pseudomonas aeruginosa* LY11 has rarely been reported. If the highly-efficient, low-cost and eco-friendly crude enzyme extract is successfully developed, it will be of great significance in bioremediation of global BDE-209 pollution.

The main objective of the present work was to study the biodegradation of BDE-209 by crude enzyme extract, focusing on the degradation efficiency and debromination efficiency of BDE-209. For comparison, biodegradation characteristics of BDE-209 by *Pseudomonas aeruginosa* LY11 were also investigated. Moreover, the biodegradation pathway and mechanism of BDE-209 were described here.

## 2. Experimental Section

### 2.1. Chemicals

BDE-209 with a purity of >99% was obtained from Alfa Aesar (Tianjin, China). Standard of BDE-209 was obtained from Sigma (St. Louis, MO, USA).

### 2.2. Strain and Culture Medium

*Pseudomonas aeruginosa* LY11, a strain for BDE-209 biodegradation used in this work, was isolated from a BFRs factory in Weifang city of Shandong province, China by our laboratory.

Two kinds of culture medium were used in this study.

A beef extract peptone medium for growing LY11 was prepared by using the following composition (1 L): beef extract 5.0 g, peptone 10.0 g and NaCl 10.0 g.

A mineral salt medium (MSM) for degradation of BDE-209 was prepared by using the following composition (1 L): CaCl_2_ 0.086 g, MgSO_4_ 0.18 g, Na_2_SO_4_ 2.0 g, KH_2_PO_4_ 5.3 g, K_2_HPO_4_ 10.6 g, KNO_3_ 10.0 g and trace elements solution 1mL [containing (g·L^−1^): FeSO_4_·7H_2_O 0.22, Co(NO_3_)_2_ 0.85, NH_4_MoO_4_ 0.83, MnCl_2_·4H_2_O 3.96, CuSO_4_·5H_2_O 5.0, ZnSO_4_·7H_2_O 5.74 and H_3_BO_3_ 1.24].

The pH of the two medium was adjusted to 7.0 by addition of HCl or NaOH before autoclaving at 121 °C for 30 min.

### 2.3. Microbial Cultivation

The strain LY11 was cultured in 500 mL Erlenmeyer flasks containing 200 mL beef extract peptone medium at 35 °C for 36 h under a rotary shaker at 200 r·min^−1^. Then, the cells were harvested and separated from the medium by centrifugation at 8000 r·min^−1^ for 10 min. Finally, the harvested cells were weighted.

Growth curve of strain LY11 under optimal incubation conditions was described by change of OD_600_ (“OD_600_”represented optical density at 600 nm) and colony-forming units (CFU) over incubation time. The values of OD_600_ were determined with an ultraviolet and visible spectrophotometer, CFU was counted via plate counting method [19].

### 2.4. Extraction of Crude Enzyme

1 g LY11 cells (age 36 h) were placed in a 50 mL centrifuge tube, suspended in the ice-cold 12 mL NaH_2_PO_4_-Na_2_HPO_4_ buffer (c = 0.05 mol/L, pH 8.5, “c” represented the buffer concentration), were subjected to 140 rounds of sonication in an ice-water bath for 3 s followed by cooling for 6 s (the time of 10 rounds was 1 min). The debris was removed by centrifugation at 12000 r·min^−1^ for 10 min. The supernatant was filtered with 0.22 μm pore-size filters and the filtrate was crude enzyme.

The protein was measured by Bradford method at 595 nm by UV-vis spectrophotometer using bovine serum albumin (BSA) as a standard [20]. 1 g LY11 cells could obtain about 100 mg crude enzyme.

### 2.5. Biodegradation System of BDE-209 under Optimal Conditions

#### 2.5.1. Biodegradation System of BDE-209 by *P. aeruginosa* LY11

One hundred milliliters of MSM and 2.0 mg BDE-209 were placed in a 250 mL Erlenmeyer flask and ultrasonically mixed by ultrasonic cleaning, 40 mg LY11 cells were added to the MSM containing BDE-209 (20 mg/L) in a super clean bench, the mixture in the flask was incubated at 35 °C on a rotary shaker at 200 rpm for 5 days.

#### 2.5.2. Biodegradation System of BDE-209 by Crude Enzyme Extract

One hundred milliliters of NaH_2_PO_4_-Na_2_HPO_4_ buffer (c = 0.05 mol/L, pH 9.0) and 2.0 mg BDE-209 were placed in a 250 mL Erlenmeyer flask and ultrasonically mixed by ultrasonic cleaning, 200 mg crude enzyme was added to the buffer containing BDE-209 (20 mg/L), the mixture in the flask was incubated at 35 °C on a rotary shaker at 200 rpm for 5 h.

### 2.6. Effects of Crude Enzyme Extract under Different Extraction Conditions

Effects of crude enzyme extract were investigated under the following extraction conditions: ultrasonic power (250–550 W), pH value (6.0–9.0), extraction time (4–16 min) and solid-liquid ratio (1:4–1:16 w/v).

### 2.7. Biodegradation Characteristics of BDE-209 under Different Conditions

#### 2.7.1. Biodegradation Characteristics of BDE-209 by *P. aeruginosa* LY11

Biodegradation characteristics of BDE-209 by LY11 were investigated under the following conditions: cell age (6–72 h), inoculation amount (10–120 mg), temperature (15–45 °C), pH value (6.0–9.0), initial concentration of BDE-209 (5–35 mg/L), biodegradation time (1–7 days).

#### 2.7.2. Biodegradation Characteristics of BDE-209 by Crude Enzyme Extract

Biodegradation characteristics of BDE-209 by crude enzyme extract were investigated under the following conditions: protein content of crude enzyme extract (100–3000 mg/L), temperature (20–50 °C), pH value (6.5–9.5), initial concentration of BDE-209 (5–35 mg/L), biodegradation time (1–7 h).

### 2.8. Extraction and Analytical Methods

After the specified incubation period, the whole samples were firstly extracted (1:1, v/v) with toluene in a separating funnel by vigorously shaking for 10 min and allowed to set until phase separation. Then, the organic phase (toluene) was collected and treated with anhydrous sodium sulfate to remove water. The aqueous phase was extracted again as described above. The combined organic phases were concentrated by using a rotary evaporator at 40 °C. Finally, the resulting residue was dissolved in 5 mL toluene for GC-MS analyses.

The quantification of BDE-209 and lower brominated PBDEs were analyzed using 7890-5975c gas chromatography-mass spectrometer (Agilent, Palo Alto, Calif. USA) equipped with a DB-5 MS column (60 m × 0.25 mm × 0.25 μm). The injection volume was 1 μL. Helium was used as the carrier gas at a flow rate of 1 mL·min^−1^. The column temperature program started at 60 °C (held for 1 min), then increased 20 °C·min^−1^ to 220 °C (held for 1 min), increased 5 °C·min^−1^ to 250 °C (held for 1 min), increased 20 °C·min^−1^ to 280 °C (held for 10 min), finally increased 15 °C·min^−1^ to 300 °C (held for 10min). Mass spectrometer conditions were: electron impact ionization, ionization energy 70 eV, full scan.

The concentration of bromide ion was measured via ICS-5000 ion chromatography (Dionex, Beijin, China) using a Dionex Ionpac AS11-HC (4 × 250 mm) analytical column (Dionex, Beijin, China). The mobile phase was 30% NaOH at a flow rate of 1.2 mL·min^−1^. The sample injection volume was 20 μL, and the column temperature was maintained at 30 °C.

### 2.9. Measurement of Degradation Efficiency and Debromination Efficiency

Biodegradation efficiency of BDE-209 and debromination efficiency were calculated according to the following Equations:

(1) $$\text{Degradation efficiency (\%)}=\frac{C_{C}-C_{S}}{C_{C}}\times 100\%$$

(2) $$\text{Debromination efficiency (\%)}=\frac{M_{Br}}{M_{T}}\times 100\%$$

where *C_C_* was initial BDE-209 concentration, *C_S_* was the BDE-209 concentration in biodegradation test; *M_Br_* was the concentration of bromide ions released, *M_T_* was the theoretical bromide ion concentration from the complete debromination of the substrate.

### 2.10. Statistical Analysis

All experiments were performed in triplicate flasks and the results presented were the mean values of the three replicates. The standard deviations for measurements ranged from 0.5% to 5.0%.

## 3. Results and Discussion

### 3.1. Effects of Crude Enzyme under Different Extraction Conditions

#### 3.1.1. Ultrasonic Power

**Figure 1:**
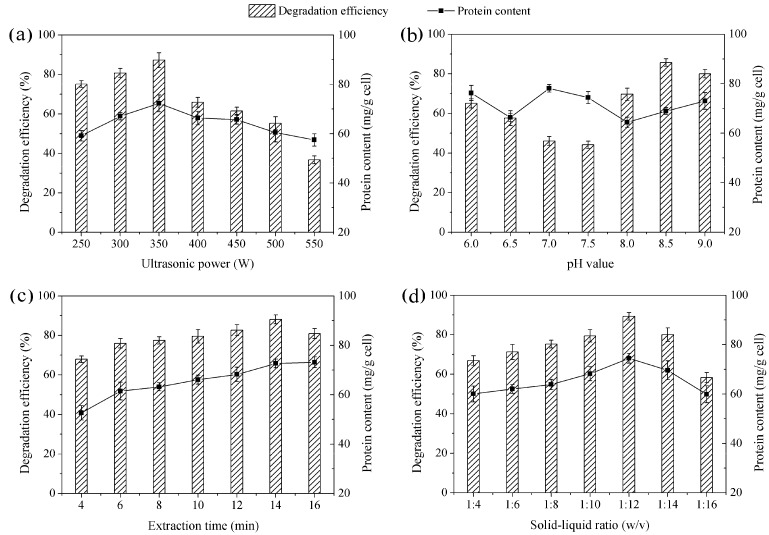
Degradation efficiency of BDE-209 and protein content of crude enzyme under different extraction conditions. (**a**) Ultrasonic power was set at 250–550 W, other conditions were set at pH 8.5, 14 min and 1:12; (**b**) pH value was set at 6.0–9.0, other conditions were set at 350 W, 14 min and 1:12; (**c**) Extraction time was set at 4–16 min, other conditions were set at 350 W, pH 8.5 and 1:12; (**d**) Solid-liquid ratio was set at 1:4–1:16, other conditions were set at 350 W, pH 8.5 and 14 min.

The effect of crude enzyme extracted at ultrasonic power ranging from 250 to 550 W is shown in Figure 1a. As shown, the crude enzyme extracted at ultrasonic power of 350 W had the highest degradation efficiency and protein content. Ultrasonic power is an important factor that would influence extraction efficiency and enzyme activity. It is well known that ultrasound-assisted extraction uses cavitation effect to extract enzyme. In general, the intensity of cavitation effect increases with increases in ultrasonic power, but cavitation effect will saturate when reaching a certain value. If ultrasonic power continues to increase, large amounts of useless cavitation bubbles will be produced. These useless cavitation bubbles increase ultrasonic scattering attenuation and decrease the intensity of cavitation effect [21]. So, in this work, extraction efficiency reached a maximum value at 350 W. Over-high ultrasonic power can instantaneously reach an atmospheric pressure in the thousands and a temperature several hundred degrees Celsius in the cavitation process. This effect can directly damage proteins and affect enzyme activity [22]. This may help theoretically explain why the degradation efficiency of BDE-209 declined sharply at an ultrasonic power of 550 W. Finally, we selected 350 W as the ultrasonic power for crude enzyme extract.

#### 3.1.2. pH Value

In general, pH value can affect enzyme activity, as different enzymes have their own optimal pH value. The effect of crude enzyme extracted at pH value ranging from 6.0 to 9.0 is shown in Figure 1b. As shown, pH 8.5 had the lower protein content, but it had a maximum value of degradation efficiency. In fact, crude enzyme extract is intracellular enzyme mixtures [23], BDE-209 degradation enzyme in crude enzyme extract still had low activity at suboptimal pH. So, BDE-209 degradation activity would be a meaningful reference for selecting an optimal pH value. As shown in Figure 1b, the crude enzyme extract was the most active at pH 8.5. Thus, pH 8.5 was selected as the ultrasonic power for crude enzyme extract.

#### 3.1.3. Extraction Time

The effect of crude enzyme extracted at different extraction times is presented in Figure 1c. It was seen that protein content of crude enzyme extract increased with an increase in extraction time. When extraction time was longer than 14 min, protein content increased slowly. Generally, increasing extraction times can improve extraction efficiency. Ultrasound can cause cell wall disruption, and long extraction time improves disruption efficiency; thus more intracellular enzyme is released to the extraction medium. However, excessive extraction time does not lead to enhanced extraction efficiency. When most of the cells have been disrupted, extraction efficiency slightly increases or no longer increases [24]. This could help us to better explain why protein content increased slowly at 16 min. While a longer extraction time can be counterproductive, more intracellular materials, except for enzyme, will be release and mixed in the crude enzyme extract [25]. These materials perhaps affected enzyme activity, thereby too long extraction time might be linked to the decline of BDE-209 degradation efficiency. For our work, 14 min appeared to be the optimal extraction time for crude enzyme extract.

#### 3.1.4. Solid-Liquid Ratio

In this study, the effect of crude enzyme extracted at above-mentioned solid-liquid ratio is shown in Figure 1d. As shown, degradation efficiency and protein content first significantly increased from 1:4 to 1:12 and then largely decreased from 1:12 to 1:16. In short, solid-liquid ratio can influence the mass transfer in the extraction medium [26]. The extraction process of crude enzyme from LY11 cell could be seen as a mass transfer process in solid-liquid phase. Cell wall was the boundary of two phases, enzyme stayed in the solid phase. When solid-liquid ratio was too low, the enzyme concentration difference at solid-liquid phase boundary was small. Driving force of diffusion was not strong enough to break down the cell wall, and this meant enzyme had a low diffusion coefficient in two phases. With solid-liquid ratio increasing, the difference in the enzyme concentration began to increase. When driving force of diffusion was strong enough to break down the cell wall, enzyme would diffuse into liquid phase, and the diffusion coefficient increased. However, when extraction efficiency of enzyme reached a maximum value, it would not increase continuously with solid-liquid ratio increasing. As shown in our work, protein content and degradation efficiency decreased at higher solid-liquid ratio than 1:12, so we selected 1:12 as the optimal solid-liquid ratio for crude enzyme extract.

Therefore, the optimal extraction conditions of crude enzyme from LY11 were ultrasonic power 350 W, pH 8.5, extraction time 14 min and solid-liquid ratio 1:12. Shi *et al.* reported that the extraction conditions of crude enzyme from *Pseudomonas aeruginosa* were ultrasonic power 450 W, pH 7.2 and extraction time 15 min [27]. It was clear that the enzyme extraction conditions in our study were different from what Shi *et al.* reported.

### 3.2. Biodegradation Characteristics of BDE-209 by *P. aeruginosa* LY11

#### 3.2.1. Cell Age

**Figure 2:**
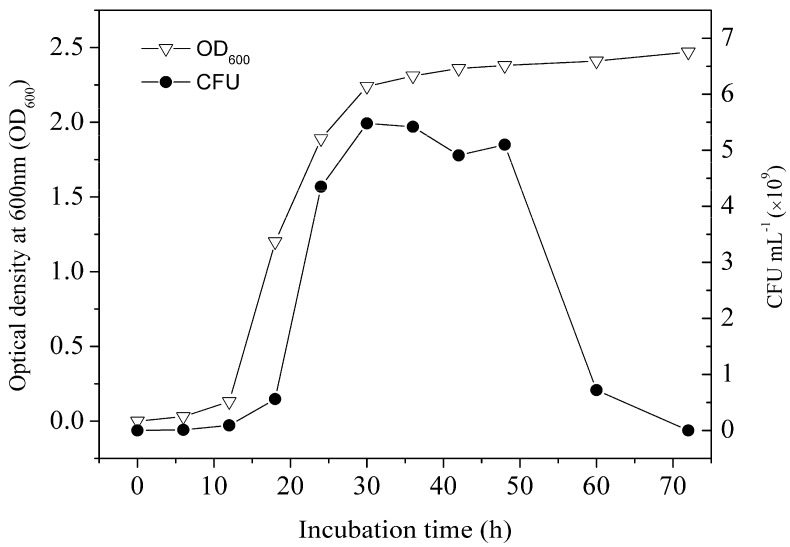
Growth curve of strain LY11 at optimal incubation conditions (temperature 35 °C, rotating speed 200 r·min^−1^, initial pH 7.0).

**Figure 3:**
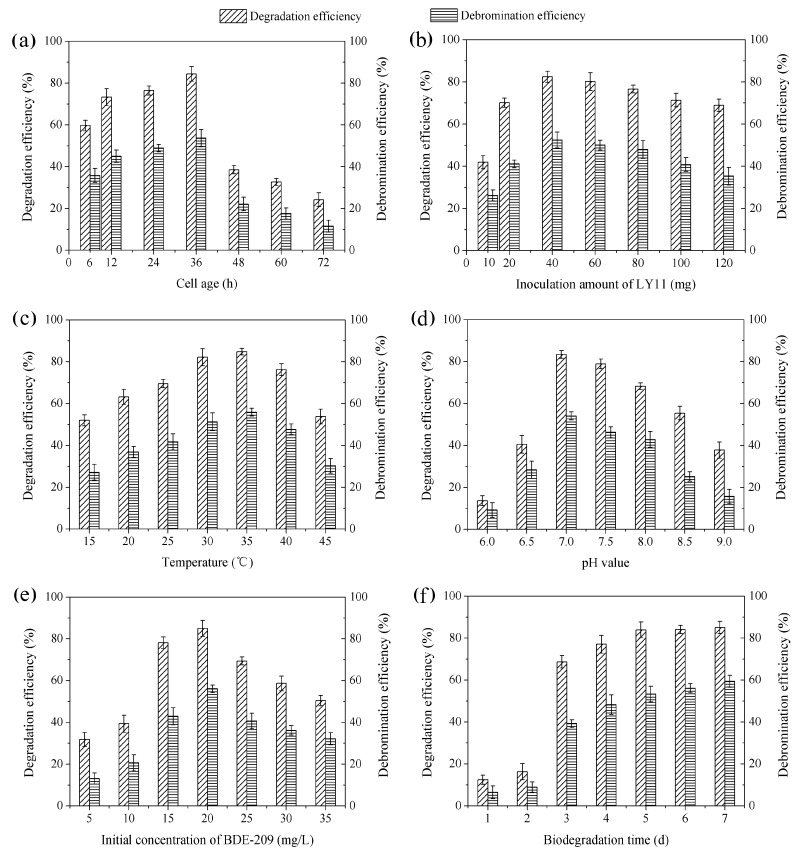
Degradation efficiency and debromination efficiency of BDE-209 by LY11 under different conditions. (**a**) Cell age was set at 6–72 h, other conditions were set at LY11 40 mg, 35 °C, pH 7.0, BDE-209 20 mg/L and 5 d; (**b**) Inoculation amount of LY11 was set at 10–120 mg, other conditions were set at age 36 h, 35 °C, pH 7.0, BDE-209 20 mg/L and 5 d; (**c**) Temperature was set at 15–45 °C, other conditions were set at age 36 h, LY11 40 mg, pH 7.0, BDE-209 20 mg/L and 5 d; (**d**) pH value was set at 6.0–9.0, other conditions were set at age 36 h, LY11 40 mg, 35 °C, BDE-209 20 mg/L and 5 d; (**e**) Initial concentration of BDE-209 was set at 5–35 mg/L, other conditions were set at age 36 h, LY11 40 mg, 35 °C, pH 7.0, and 5 d; (**f**) Biodegradation time was set at 1–7 d, other conditions were set at age 36 h, LY11 40 mg, 35 °C, pH 7.0 and BDE-209 20 mg/L.

Growth curve of strain LY11 is shown in Figure 2. From the growth curve shown in Figure 2, four stages (lag phase, logarithmic growth phase, stationary phase and decline phase) were clearly observed. The incubation time represents the cell age of LY11. As far as we know, if there is no direct carbon to use in the growing environment, bacteria will produce some enzymes to degrade organics as a sole carbon and energy source [28]. Based on our work, cell age of 12–24 h occurred in mid-logarithmic growth phase, and cells had produced a certain amount of BDE-209 degradation enzymes and possessed a higher enzyme activity in this phase. As shown in Figure 3a, cell ages of 12 and 24 h exhibited higher degradation efficiency and debromination efficiency. Nevertheless, cell age of 36 h occurred in the late-logarithmic growth phase, whereby cells had produced the maximum amount of BDE-209 degradation enzymes and possessed the highest enzyme activity in this phase. Accordingly, cell age of 36 h exhibited the highest degradation efficiency and debromination efficiency. While at cell age of 48–72 h, cells began to enter into stationary phase and decline phase. In the two phases, cells stopped growing, metabolism also became slow, and enzyme activity decreased remarkably. Hence, we chose cell age of 36 h as the optimal condition of BDE-209 biodegradation by LY11.

#### 3.2.2. Inoculation Amount of LY11

As shown in Figure 3b, a significant increase of degradation efficiency and debromination efficiency was observed at inoculation amount of 10–40 mg, and a slight decline was observed at the inoculation amount of 60–120 mg. This phenomenon can be well explained by the bacterial competition mechanism [29]. When the inoculation amount was small, per unit mass of cells had large amount of BDE-209 to use. Hence, degradation efficiency and debromination efficiency increased with an increasing inoculation amount, and reached a maximum value at 40 mg. When the inoculation amount continued to increase, per unit mass of cells did not have enough BDE-209 to use, and competitive inhibition occurred. Competitive inhibition could inhibit cell growth and enzyme activity, and had a more significant inhibition with inoculation amount increasing. This could explain why degradation efficiency and debromination efficiency decreased at more than 40 mg. Therefore, an inoculation amount of 40 mg seemed to be the best condition for BDE-209 biodegradation by LY11.

#### 3.2.3. Temperature

Temperature plays a vital role in organics degradation due to its influence on enzyme activity. At a suitable temperature, cells can grow quickly and produce high amount of enzymes, thus having enzyme activity for organics degradation [30]. Figure 3c shows the effect of BDE-209 biodegradation at different temperatures. As shown, degradation efficiency and debromination efficiency reached a maximum value at 35 °C. It suggested that LY11 had the highest enzyme activity for BDE-209 biodegradation at 35 °C. Consequently, we chose temperature of 35 °C as the optimal condition for BDE-209 biodegradation by LY11.

#### 3.2.4. pH Value

In bacteria, pH can influence nutrient absorption and enzyme production [31]. At optimal pH, LY11 could absorb more BDE-209 and produce more enzymes for BDE-209 biodegradation. Conversely, at suboptimal pH, LY11 could not absorb enough BDE-209 for growing. As shown in Figure 3d, neutral pH 7.0 had the highest degradation efficiency and debromination efficiency. It illustrated LY11 owned the best effect of biodegradation at pH 7.0, and we chose pH 7.0 as the optimal condition for BDE-209 biodegradation.

#### 3.2.5. Initial Concentration of BDE-209

The effect of BDE-209 biodegradation at different initial concentrations is presented in Figure 3e. It was seen that degradation efficiency and debromination efficiency had a maximum value at initial concentration of 20 mg/L. This means that initial concentration of 20 mg/L was the optimal condition. However, low initial concentrations such as 5 and 10 mg/L, exhibited the lower degradation efficiency and debromination efficiency. This might due to too low initial concentrations of BDE-209 that did not support cell growth and induced less degradation enzyme, which led to low bioavailability of BDE-209. When initial concentration of BDE-209 was increasing, it increased collision probability with cells, and induced more degradation enzyme. However, degradation efficiency and debromination efficiency decreased at more than 20 mg/L. This was because too high initial concentrations of BDE-209 could also inhibit cell growth.

#### 3.2.6. Biodegradation Time

Figure 3f shows the effect of BDE-209 biodegradation at different times. As shown, BDE-209 biodegradation by LY11 had a lag period in the first 2 days, and then entered a rapid degradation period from 3 to 5 days, finally reaching a steady period after 5 days. In a general way, bacteria have an adaptation process to xenobiotic organic compounds when there is no direct carbon for growing. In this period, bacteria begin to produce degradation enzyme for degrading these xenobiotics; thus this period has very low degradation efficiency. Nevertheless, when degradation enzyme production accumulates to a certain amount, degradation efficiency begins to increase remarkably [32]. This could help explain why during the first 2 days there was a lag period and 3–5 days there was a rapid degradation period in our work. Considering the slight increase of BDE-209 degradation at a biodegradation time longer than 5 days, we finally selected the biodegradation time of 5 days as the optimal condition for BDE-209 biodegradation by LY11.

In this part, the optimal conditions for BDE-209 biodegradation by LY11 were cell age 36 h, inoculation amount 40 mg, temperature 35 °C, pH 7.0, BDE-209 20 mg/L and biodegradation time 5 days. Shi *et al.* had reported that the degradation rate of BDE-209 by *Pseudomonas aeruginosa* was 57%, its biodegradation conditions were cell age 24 h, temperature 30 °C, pH 7.3, BDE-209 1 mg/L and biodegradation time 6 days [16]. In contrast, LY11 exhibited markedly better bio-removal ability of BDE-209 than the strain reported by Shi *et al.* [16].

### 3.3. Biodegradation Characteristics of BDE-209 by Crude Enzyme Extract

#### 3.3.1. Protein Content of Crude Enzyme Extract

**Figure 4:**
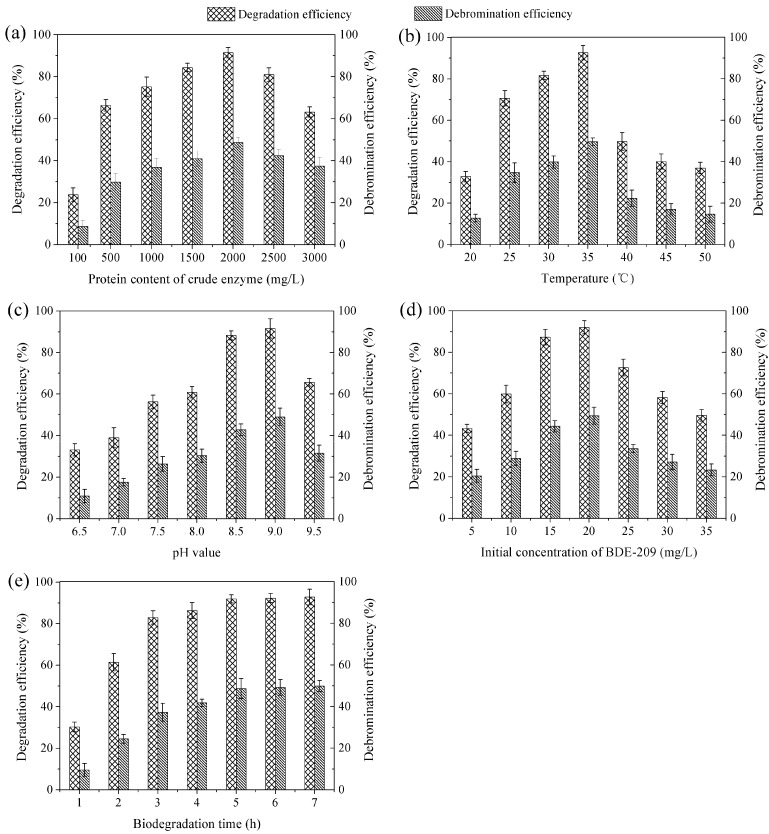
Degradation efficiency and debromination efficiency of BDE-209 by crude enzyme extract under different conditions. (**a**) Protein content of crude enzyme was set at 100-3000 mg/L, other conditions were set at 35 °C, pH 9.0, BDE-209 20 mg/L and 5 h; (**b**) Temperature was set at 20–50 °C, other conditions were set at crude enzyme 2000 mg/L, pH 9.0, BDE-209 20 mg/L and 5 h; (**c**) pH value was set at 6.5–9.5, other conditions were set at crude enzyme 2000 mg/L, 35 °C, BDE-209 20 mg/L and 5 h; (**d**) Initial concentration of BDE-209 was set at 5–35 mg/L, other conditions were set at crude enzyme 2000 mg/L, 35 °C, pH 9.0 and 5 h; (**e**) Biodegradation time was set at 1–7 h, other conditions were set at crude enzyme 2000 mg/L, 35 °C, pH 9.0 and BDE-209 20 mg/L.

As mentioned earlier, crude enzyme extract consisted of intracellular enzyme mixtures, and the BDE-209 degradation enzyme in it played a leading role in biodegradation. So far, we have not been able to determine the content of the BDE-209 degradation enzyme accurately, because we have not known which enzyme had the ability of BDE-209 degradation in the crude enzyme extract. Because the content of BDE-209 degradation enzyme was in direct proportion to crude enzyme extract, we determined the content of crude enzyme extract instead of BDE-209 degradation enzyme. So, we determined degradation efficiency and debromination efficiency at amounts of protein of crude enzyme extract, and these results are shown in Figure 4a. As can be seen in Figure 4a, protein content of 2000 mg/L exhibited the highest value of BDE-209 biodegradation. This indicated that protein content of 2000 mg/L was the best condition for BDE-209 biodegradation by crude enzyme extract. Meanwhile, low degradation efficiency and debromination efficiency were detected at low protein content of crude enzyme extract, due to the lower content of BDE-209 degradation enzyme. Nevertheless, degradation efficiency and debromination efficiency began to decrease when the protein content was higher than 2000 mg/L, caused by too much protein. If a high amount of protein of crude enzyme extract was put into the biodegradation system, too much enzyme which possessed no activity for BDE-209 degradation reduced the probability of collisions between BDE-209 and its degradation enzyme.

#### 3.3.2. Temperature

As a biocatalyst, enzyme exhibits temperature effect just like catalyst. Temperature increment can increase enzymatic reaction velocity. However, on the other hand, it also can accelerate enzyme denaturation velocity. Therefore, in the low temperature range, enzymatic reaction velocity increases with temperature increasing. However, above a certain temperature, enzymatic reaction velocity begins to decrease [33]. As shown in Figure 4b, degradation efficiency and debromination efficiency increased in the low temperature range of 20–35 °C, and decreased with increasing temperature above 35 °C. This phenomenon was in accord with the explanation mentioned above. Accordingly, a temperature of 35 °C was the optimum temperature for BDE-209 biodegradation by crude enzyme extract.

#### 3.3.3. pH Value

As explained above, pH value has a strong influence on enzyme activity. At a certain pH value, enzyme will exhibit the maximum catalytic activity, this pH is the optimum pH value for enzyme. Enzyme activity is low or undetectable at pH value far from the optimum pH value. Already from results presented in Figure 4c it was obvious that pH 9.0 obtained the highest value of degradation efficiency and debromination efficiency. Thus, pH 9.0 was the optimum pH value for BDE-209 biodegradation by crude enzyme extract.

#### 3.3.4. Initial Concentration of BDE-209

At different initial concentrations of BDE-209, the biodegradation behaviour of crude enzyme extract is displayed in Figure 4d. As seen from Figure 4d, degradation efficiency and debromination efficiency increased at first with initial concentration increasing and then decreased, the maximum values were at 20 mg/L. This might be due to the fact that too low initial concentration resulted in low collision probability between degradation enzyme and BDE-209. If initial concentration continued to increase, collision probability would increase evidently. However, when almost all degradation enzymes were involved in a reaction with BDE-209, collision probability did not continue to increase, and degradation efficiency decreased because there were not enough enzymes to degrade excessive BDE-209. Therefore, initial concentration of 20 mg/L was selected as the optimum concentration for BDE-209 biodegradation by crude enzyme extract.

#### 3.3.5. Biodegradation Time

As presented in Figure 4e, degradation efficiency and debromination efficiency increased quickly from 1 to 5 h, but increased slightly after 5 h. Most likely, this phenomenon was also related to collision probability between degradation enzyme and BDE-209. The short biodegradation time gave rise to low collision probability. Likewise, collision probability would increase with biodegradation time. However, most degradation enzymes had already reacted with BDE-209 when collision probability reached a certain value, it would slightly increase after that value. So, it had been suggested that at 5 h the higher biodegradation value was first reached, and we finally chose it as the optimum time for BDE-209 biodegradation by crude enzyme extract.

Obviously, the optimal conditions for BDE-209 biodegradation by crude enzyme extract from LY11 were: protein content 2000 mg/L, temperature 35 °C, pH 9.0, BDE-209 20 mg/L and biodegradation time 5 h. Shi *et al.* had reported that the degradation rate of BDE-209 by crude enzyme obtained from *Pseudomonas aeruginosa* was 69%, its biodegradation conditions were temperature 30 °C, pH 7.3, BDE-209 1 mg/L and biodegradation time 12 h [16]. These results suggested that crude enzyme extract from LY11 degraded a higher concentration of BDE-209 in a shorter time than crude enzyme reported by Shi *et al*. Nevertheless, environmental application of BDE-209 bio-removal by crude enzyme extract will be a key research focus in the future. As shown in Figure 4b, in the normal environmental temperatures (20–35 °C), degradation efficiency ranged from 32.82% to 92.63%, and debromination efficiency ranged from 12.77% to 49.68%. As shown in Figure 4c, in the normal environmental pH (6.5–8.5), degradation efficiency ranged from 33.14% to 88.28%, and debromination efficiency ranged from 10.91% to 42.73%. This indicated that temperature and pH were the most important parameters affecting degradation efficiency and debromination efficiency of BDE-209 when crude enzyme extract was applied.

### 3.4. The Contrast Biodegradation of BDE-209 between LY11 and Crude Enzyme Extract under Optimal Conditions

At the optimal conditions selected above, the biodegradation effects of LY11 and crude enzyme extract are listed in Table 1. As listed in Table 1, degradation efficiency of crude enzyme extract (92.77%) was higher than LY11 (85.12%), but debromination efficiency of crude enzyme extract (49.86%) was lower than LY11 (56.03%). I think it was probably because LY11 preferred to use lower brominated PBDEs which were produced as a result of BDE-209 biodegradation, and the lower brominated PBDEs came into competition with BDE-209. Nevertheless, most of the degradation enzymes in crude enzyme extract could combine and react with BDE-209 in a short time (5 h), and there were not enough degradation enzymes to further debrominate. While LY11 was a living cell, it had no choice but to continually decompose lower brominated PBDEs as its sole organic carbon source over a long time (5 days). So, it was reasonable for LY11 to possess the lower degradation efficiency and higher debromination efficiency. Hence, the results presented here clearly demonstrate that crude enzyme extract had a satisfactory result for BDE-209 biodegradation. Most importantly, it demonstrated the shortest biodegradation time as far as we know. Certainly, it is necessary to conduct an in-depth study to improve its lower debromination efficiency in the future work.

**Table 1:** The contrast biodegradation of BDE-209 between *P. aeruginosa* LY11 and crude enzyme extract under optimal conditions. The optimal biodegradation condition of LY11 was set at cell age 36 h, inoculation amount 40 mg, initial concentration 20 mg/L, 35 °C, pH 7.0 and 5 days. The optimal biodegradation condition of crude enzyme extract was set at protein content 2000 mg/L, initial concentration 20 mg/L, 35 °C, pH 9.0 and 5 h.

Degradation Sample	Degradation Efficiency (%)	Debromination Efficiency (%)
*P. aeruginosa* LY11	85.12 ± 1.13 ^a^	56.03±1.37 ^b^
Crude Enzyme Extract	92.77 ± 0.51 ^a^	49.86±0.85 ^a^

^a^ *p* < 0.05, ^b^ *p* < 0.01.

### 3.5. Biodegradation Mechanism of BDE-209 by Crude Enzyme Extract

**Figure 5:**
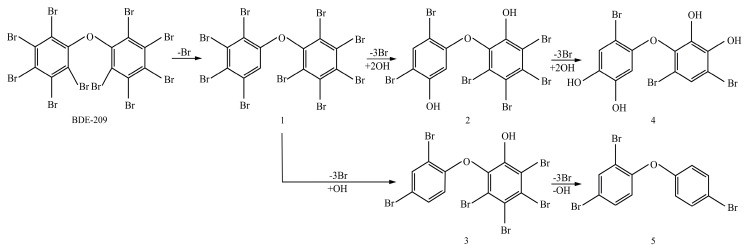
A possible BDE-209 biodegradation pathway by crude enzyme extract. 1–5 were possible BDE-209 biodegradation products listed in Table 2.

After being reacted with crude enzyme extract for 5 h, BDE-209 biodegradation products were analyzed using the above method mentioned in Section 2.8. The chemical structures and names of BDE-209 biodegradation products are listed in Table 2. As shown in Table 2, BDE-209 biodegradation products detected by the GC-MS analysis mainly were lower brominated PBDEs and OH-PBDEs. To get a better insight into the fate of BDE-209, the possible biodegradation pathway and mechanism were proposed based on the biodegradation products (Figure 5). As shown in Figure 5, BDE-209 was initially debrominated, losing one bromine atom to form BDE-206 (1). Then, BDE-206 was further debrominated and had two likely pathways. In the first pathway, BDE-206 lost three bromine atoms and added two hydroxyl groups to successively generate 2′,5-dihydroxy-2,3′,4,4′,5′,6′-hexabromodiphenyl ether (2) and 2′,3′,4,5-tetrahydroxy-2,4′,6′-tribromodiphenyl ether (4). In the second pathway, BDE-206 first removed three bromine atoms and added one hydroxyl group to yield 6-OH-BDE-137 (3). Afterwards, 6-OH-BDE-137 continued to remove three bromine atoms and one hydroxyl group, which resulted in forming BDE-28 (5).

**Table 2:** Possible chemical structures of BDE-209 biodegradation products identified by GC-MS analysis in the biodegradation system of crude enzyme extract.

Number	Molecular Weight	Biodegradation Products	Possible Chemical Structure
1	881	2,2′,3,3′,4,4′,5,5′,6′-Nonabromodiphenyl ether (BDE-206)	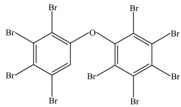
2	676	2′,5-Dihydroxy-2,3′,4,4′,5′,6′-hexabromodiphenyl ether	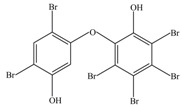
3	660	2′-Hydroxy-2,3′,4,4′,5′,6′-hexabromodiphenyl ether (6-OH-BDE-137)	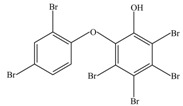
4	471	2′,3′,4,5-Tetrahydroxy-2,4′,6′-tribromodiphenyl ether	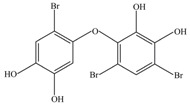
5	407	2,4,4′-Tribromodiphenyl ether (BDE-28)	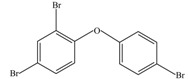

It was easy to establish a general rule of BDE-209 biodegradation by crude enzyme extract. First of all, BDE-209 lost one bromine atom to produce BDE-206. Then, BDE-206 successively removed three bromine atoms and was accompanied by hydroxylation reaction to produce lower brominated PBDEs and OH-PBDEs. It was noteworthy that 6-OH-BDE-137 was one of BDE-209’s biodegradation products. Nevertheless, 6-OH-BDE-137 is a common OH-PBDE which has been widely detected in the environment and tissues of wildlife, and has been attracting growing concern by researchers owing to its potential toxicity [34]. Furthermore, the formation of OH-PBDEs has also received increasing attention in recent years, as they have been reported in both humans and wildlife, and there is growing evidence that OH-PBDEs have potential to disrupt the endocrine system and oxidative phosphorylation [35,36]. Thus, BDE-209 biodegradation mechanism proposed above provided a likely formation pathway of OH-PBDEs in the environment. Our next step would be to conduct an intensive study of OH-PBDEs’ formation and biodegradation mechanism by enzymes. However, incomplete debromination was presented in Figure 5, and this could explain why crude enzyme extract possessed the lower debromination efficiency of BDE-209 than LY11. Furthermore, the lowest brominated PBDE in products was BDE-28. This is usually of great concern, due to its extensive occurrence, bioaccumulation and unfavorable effects [37]. So, we would need to research BDE-28 biodegradation by crude enzyme extract in the future. In summary, the BDE-209 biodegradation mechanism of this study has laid the foundation for further investigations to exploit efficient enzymes for complete debromination of BDE-209. It also helped us to understand the biodegradation process of BDE-209 in the environment and was instructive for controlling increasingly serious PBDE pollution.

To the authors’ knowledge, this is the first report on the biodegradation of BDE-209 by crude enzyme extract from *P. aeruginosa* LY11. These results may provide substantial support for the potential application of crude enzyme in bioremediation of heavy BDE-209 pollution in the environment. The future work will focus on the genes that encode the BDE-209 degradation enzyme and the complete debromination mechanism of the BDE-209 degradation enzyme.

## 4. Conclusions

In the present study, the biodegradation of BDE-209 by crude enzyme extract from *P. aeruginosa* LY11 showed very encouraging results, its degradation efficiency reaching 92.77% after 5 h. By contrast to *P. aeruginosa* LY11, crude enzyme extract exhibited obviously higher degradation efficiency and shorter biodegradation time. BDE-209 biodegradation products by crude enzyme extract were detected, and its pathway was also tentatively proposed. The biodegradation mechanism was presented that crude enzyme extract could degrade BDE-209 into lower brominated PBDEs and OH-PBDEs through debromination and hydroxylation of the aromatic rings.
